# Chemical Composition and Cytotoxic Activity of the Fractionated Trunk Bark Essential Oil from *Tetraclinis articulata* (Vahl) Mast. Growing in Tunisia

**DOI:** 10.3390/molecules26041110

**Published:** 2021-02-19

**Authors:** Salma Jlizi, Aida Lahmar, Afifa Zardi-Bergaoui, Roberta Ascrizzi, Guido Flamini, Abdel Halim Harrath, Leila Chekir-Ghedira, Hichem Ben Jannet

**Affiliations:** 1 Laboratory of Heterocyclic Chemistry, Natural Products and Reactivity (LR11ES39), Medicinal Chemistry and Natural Products Team, Faculty of Science of Monastir, University of Monastir, Avenue of Environment, 5019 Monastir, Tunisia; salma.jlizi@gmail.com (S.J.); afifa_zardi@hotmail.fr (A.Z.-B.); 2 Bioactive Natural Products and Biotechnology Research UnitUR17ES49, Faculty of Dental Medicine of Monastir, University of Monastir, Avicenne Street, 5000 Monastir, Tunisia; elahmar.aida@gmail.com (A.L.); leila.chekir@laposte.net (L.C.-G.); 3 Dipartimento di Farmacia, Università di Pisa, Via Bonanno 6, 56126 Pisa, Italy; roberta.ascrizzi@gmail.com (R.A.); guido.flamini@farm.unipi.it (G.F.); 4 Centro Interdipartimentale di Ricerca “Nutraceutica e Alimentazione per la Salute”, Università di Pisa, Via del Borghetto 80, 56124 Pisa, Italy; 5 Department of Zoology, College of Science, King Saud University, Riyadh 11564, Saudi Arabia; halim.harrath@gmail.com

**Keywords:** *Tetraclinis articulata*, trunk bark, essential oil, fractionation, chemical composition, cytotoxic activity

## Abstract

The aim of the present research was to determine the chemical composition and the cytotoxic effects of *Tetraclinis articulata* trunk bark essential oil (HEE) obtained by steam distillation and five fractions obtained by normal phase silica chromatographic separation. Chemical analysis allowed the identification of 54 known compounds. Relatively high amounts of oxygenated sesquiterpenes (44.4–70.2%) were detected, mainly consisting of caryophyllene oxide (13.1–26.6%), carotol (9.2–21.2%),14-hydroxy-9-*epi*-(*E*)-caryophyllene (3.2–15.5%) and humulene epoxide II (2.6–7.2%). The cytotoxic activity against human mammary carcinoma cell lines (MDA-MB-231) and colorectal carcinoma cell lines (SW620) of the essential oil and its fractions were assessed. All the samples displayed moderate to weak activity compared to 5-fluorouracil. The colorectal carcinoma cell line was relatively more sensitive to the essential oil and its fractions compared to the breast cancer cell line, showing IC_50_ values from 25.7 to 96.5 μg/mL. In addition, the essential oil and its fraction E.2 revealed a cytotoxic activity against colorectal carcinoma cell line, with IC_50_ values lower than 30 μg/mL. This is the first report on the chemical composition and cytotoxic activity of the trunk bark essential oil of *T. articulata*.

## 1. Introduction

Medicinal and aromatic plants (MAPs), in their entirety or their separated parts, are used as they are or can be further processed by extraction of essential oils and are considered an important resource in various fields, such as pharmaceutical, flavor and fragrance, perfumery, and cosmetic industries [1]. Essential oils from medicinal and aromatic plants are a very interesting source of secondary metabolites because of their many different biological properties, such as cytotoxic [2], anti-inflammatory [3], antioxidant [4], insecticidal [5], antifungal [6] and antimicrobial [7,8,9], many of which are of increasing interest in the field of human and animal health. In recent years, the development of antibiotic resistance represents the major issue in medical microbiology and the search for new weapons against antibiotic resistances has led to the search of new sources of potential antimicrobials. Among them, the plant kingdom offers a wide range of biodiversity with a great value for the pharmaceutical industry. In Tunisia and in other North African countries, MAPs in human and veterinary medicine are mainly used for respiratory and intestinal infections in children, and in the treatment of gastric pains, diabetes, hypertension, diarrhea and rheumatism [10,11,12,13]. The genus *Tetraclinis* is part of the Cupressaceae family and unspecific, consisting of only *Tetraclinis articulata* (Vahl) Masters (synonyms: *Thuja articulata* Vahl, *Callitris quadrivalvis* Vent), commonly known as “Barbary thuja”. This species is widespread across the North African region. In Tunisia, this coniferous tree is located in the northeastern region of the country [14]. Previous studies on this species indicated its use as an antibacterial and antifungal [13,15,16], cytotoxic [17], antioxidant and anti-inflammatory agent [18]. The essential oils isolated from some *Tetraclinis articulata* organs (nonwoody branches, wood branches, wood, cones, roots, leaves, fruits and seeds) so far have exhibited wide variability in their major compounds, the most commonly encountered ones being monoterpene hydrocarbons, such as α-pinene in leaves, fruits and cones (Algeria and Tunisia) [19,20,21] and camphene in wood branches and roots (Tunisia) [22], followed by oxygenated monoterpenes such as camphor and bornyl acetate in roots, leaves, nonwoody branches and wood branches (Malta, Algeria and Tunisia) [16,23,24].To date, no reports have been published on the chemical and cytotoxic activity of the trunk bark essential oil of *T. articulata* (HEE). Our aim was thus to isolate the essential oils of HEE from northeast Tunisia by steam distillation, and fractionate these by normal phase silica chromatography and assess the fractions for cytotoxic activity [25].

## 2. Results and Discussion

### 2.1. Chemical Composition of the Trunk Bark Essential Oil (HEE) and Its Fractions (E.1–E.5)

**Figure 1:**
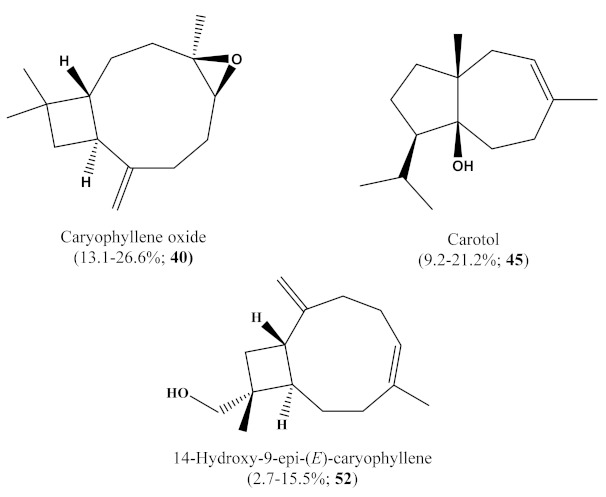
Chemical structures of some major compounds identified in *Tetraclinis articulata* trunk bark essential oil (HEE) and its fractions (E.1–E.5).

The steam distillation of the trunk bark of *T. articulata* yielded 0.05% (*w*/*w*) of a pale-yellow colored oil. The full chemical profiles were determined by Gas Chromatography coupled to Mass Spectrometry (GC/MS) and are presented in Table 1. Overall, 54 compounds were identified, representing from 90.8% up to 96.0% of the total compositions. Terpenes (in the form of both mono- and sesquiterpenes) were detected as the main chemical group of compounds, with relative concentrations ranging from 88.2% to 95.8%. Oxygenated sesquiterpenes were the most abundant chemical class of compounds in all samples. Among them, caryophyllene oxide (**40**) was detected as the major constituent in all samples, ranging from a minimum of 13.1% in HEE, up to 26.6% in E.2, carotol (**45**) and 14-hydroxy-9-*epi*-(*E*)-caryophyllene (**52**) followed, with relative percentages varying from a minimum of 9.2% in HEE to a maximum of 21.2% in E.4, and between 2.7% in E.1 and 15.5% in E.5. Oxygenated monoterpenes were the second most abundant chemical class in HEE and E.1; α-terpineol (0.3–10.4%; **12**), borneol (0.3–4.1%; **9**), and geranyl 2-methylbutyrate (0.2–4.9%; **41**) were the most represented. In all other samples, sesquiterpene hydrocarbons followed as the second most abundant chemical group, among which α-muurolene (**30**, from 5.5% in HEE, up to 12.5% in E.5) and β-caryophyllene (**24**, not detected in E.5, up to 5.5% in HEE) showed the highest relative presence. Caryophyllene oxide, β-caryophyllene and carotol (Figure 1) have all been described as potent medicinal compounds with various activities, such as analgesic [26] and cytotoxic [27]. To the best of our knowledge, this is the first report on the itemized chemical characterization of the trunk bark essential oil of *T. articulata*. In spite of the many investigations into the chemical composition of essential oils from the different organs of the same species, the trunk bark essential oil of *T. articulata* presented a totally distinct chemical profile. In Tunisia, Tekaya-Karoui et al. [16,22] demonstrated that the main components in the nonwoody branches oil was *Z*-muurolene (29.0%) and 4,6-dimethyl-octane-3,5-dione (22.4%). In the cone oil, the most important compounds found were *p*-cymene-8-ol (10.4%) and β-phellandrene (8.1%). The major constituent in the oils of the roots and the woody branches was found to be camphene (70.2 and 43.2%, respectively). Nonan-1-ol was the main constituent present in the fraction of the essential oil from woody terminal branches (75.2%).In the fraction of the essential oil from roots, the major compound detected was bornyl acetate (16.6%).The results reported by Barrero et al. [12], from Morocco, have shown that the leaf essential oil was rich in bornyl acetate (16.5%), camphor (19.1%) and borneol (9.6%), and that the essential oil from the wood was rich in cedrol (28.2%) and 1,7-di-*epi*-cedrol (17.9%).

### 2.2. Cytotoxic Activity

**Figure 2:**
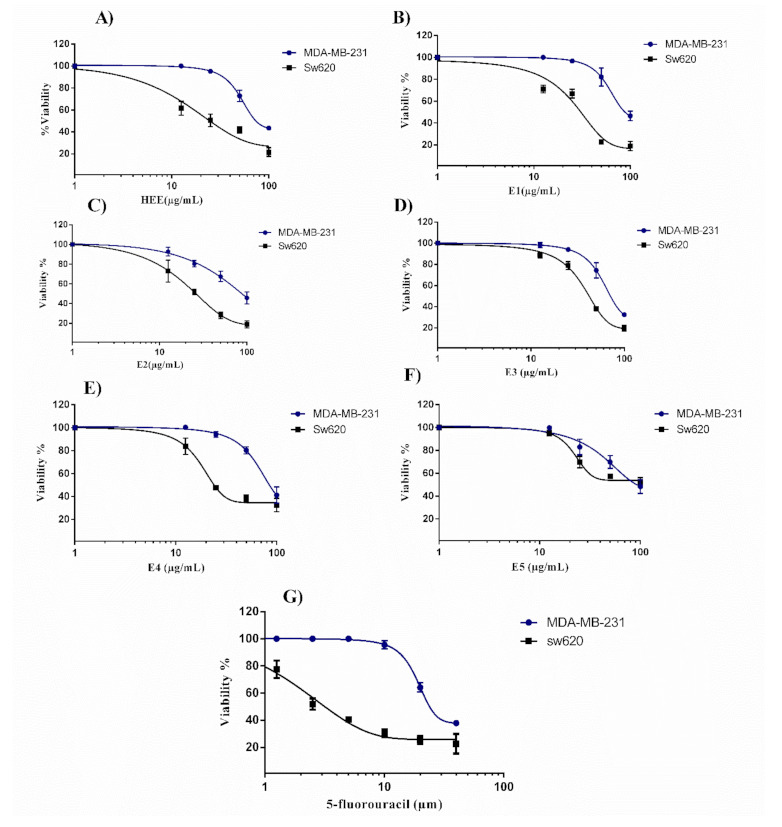
Inhibitory effects on MDA-MB-231 and SW620 cell proliferation with increasing concentrations (0–100 mg/mL) for 48 h of (**A**) *T. articulata* trunk bark complete essential oil (HEE); (**B**) Fraction E.1; (**C**) Fraction E.2; (**D**) Fraction E.3; (**E**) Fraction E.4; (**F**) Fraction E.5; (**G**) 5-fluorouracil. The percentage of cell viability was determined using crystal violet assay. Results are expressed as mean percentage of control growth ± SD of three independent experiments.

To determine whether *T. articulata* trunk bark essential oil and its fractions could exert a cytotoxic action, we exposed MDA-MB-231 breast cancer cells and SW620 colon cancer cells to increasing amounts of tested agents (0 to 100 µg/mL) for 48 h, prior to a cell viability assay (Table 2, Figure 2). A dose-dependent cytotoxic activity was shown against both cell lines with the SW620 cells being more sensitive to all tested fractions. Several reports highlighted the expression of multidrug resistance proteins in MDA-MB-231 cell line [28,29]. The complete essential oil of *T. articulata* displayed the highest inhibitory activity against SW620 and MDA-MB-231 cells with IC_50_ values of 25.7 and 83.0 µg/mL, respectively. Interestingly, fraction E.2 was most active against SW620 cells and E.3 was most active against MDA cells. The essential oil of *T. articulata* trunk bark, and its fractions, showed low cytotoxicity towards both cell lines, compared to the positive control used. According to American National Cancer Institute, however, extracts with IC_50_ values below 30 μg/mL against experimental cancer cell lines may represent promising anticancer agents for further drug development [30].

The cytotoxic activity of the essential oil of *T. articulata* trunk bark and its fractions may be attributed to specific components of the oils. In previous works [27,31,32,33], it has been shown that caryophyllene oxide (HEE: 13.1%, E.1: 25.2%, E.2: 26.6%, E.3: 20.5%, E.4: 20.2% and E.5: 21.9%; **40**) and carotol (HEE: 9.2%, E.1: 12.0%, E.2: 20.3%, E.3: 20.5%, E.4: 21.2% and E.5: 15.1%; **45**) (Table 1), which are predominant in the essential oil and its fractions, could be responsible for the cytotoxic activity. It has been reported that caryophyllene oxide inhibited cell growth, in a dose-dependent and cell-specific manner, against different types of cell lines, e.g., HepG2, AGS, HeLa, SNU-1 and SNU-16 cells, with IC_50_ values ranging from 3.9 to 27.4 µM [34]. Therefore, the cytotoxicity of the crude essential oil and its fractions could be due to these sesquiterpenes, as already reported for the cytotoxic activity of *Myrica gale* L. essential oil [35]. In addition, minor components could also contribute to cytotoxic activity of the oils, acting in synergy with the other quantitatively predominant compounds [36,37].

## 3. Material and Methods

### 3.1. Plant Material

The trunk bark of *T. articulata* was collected from the region of Mornag, Governorate of Ben Arous (northeast Tunisia), in January 2019. The plant material identification was accomplished by Professor F. Harzallah-skhiri (Higher Institute of Biotechnology of Monastir, University of Monastir, Tunisia). A voucher specimen (TA-19) was deposited at the Laboratory of Heterocyclic Chemistry, Natural Products and Reactivity (LR11ES39), Faculty of Sciences of Monastir, Tunisia, for further reference.

### 3.2. Isolation and Fractionation of the Essential Oil

Trunk bark of *T. articulata* (750 g) was cut into little pieces and subjected to steam distillation over 4 h using a Clevenger-type system. The essential oil (HEE) obtained was decanted, dried over anhydrous Na_2_SO_4_ and stored in sealed glass vials at 4–5 °C until chemical and biological analysis. The essential oil (300 mg) was fractionated on a column of silica gel using a hexane/ethyl acetate step gradient (95:5; 90:10; 80:20; 70:30) to afford five fractions (E.1–E.5): fraction E.1 (137 mg, 45.7% of oil); fraction E.2 (37 mg, 12.4% of oil); fraction E.3 (28 mg, 9.4% of oil); fraction E.4 (22 mg, 7.4% of oil); fraction E.5 (46 mg, 15.4% of oil). These fractions (E.1–E.5) were also submitted to gas chromatography coupled with mass spectrometry.

### 3.3. Gas Chromatography–Mass Spectrometry Analyses and Peak Identification

The composition of HEE and its five fractions was determined by Gas Chromatography/Electron Ionization-Mass Spectrometry (GC-EI-MS). Analyses were carried out with a Varian CP-3800 gas chromatograph (Varian Inc., Palo Alto, CA, USA) fitted with an HP-5 capillary column (30 m, 0.25 mm, 0.25 μm film thickness) coupled with a Varian Saturn (Varian Inc., Palo Alto, CA, USA) 2000 ion-trap mass detector. Operating conditions were as follows: injector temperature, 220 °C; transfer line temperature, 240 °C; oven temperature, 60 to 240 °C (set to a 3 °C/min increment), carrier gas: helium at a 1 mL/min flow. After dilution (5%) in HPLC-grade *n*-hexane, 1 µL was injected in the GC (split ratio 1:30). The acquisition was performed with the following parameters: full scan, with a scan range of 35–300 *m*/*z*; scan time: 1.0 s; threshold: 1 count. The identification of the constituents was based on the comparison of their retention times (t_R_) with those of pure reference samples and of their linear retention indices (LRIs), which were determined relative to the t_R_ of a series of *n*-alkanes (C_9_–C_25_). The mass spectra detected were compared with those listed in the commercial libraries NIST 14 and ADAMS, and in a homemade mass-spectral library, built from pure substances and components of essential oils of known composition and MS literature data [38,39].

### 3.4. Cell Lines

Human mammary carcinoma cell line (MDA-MB-231) and colorectal carcinoma cell line (SW620) were obtained from the American Tissue Culture Collection (ATCC, Molsheim, France). Cells were maintained in a 5% CO_2_ humidified atmosphere at 37 °C and cultured in Roswell Park Memorial Institute (RPMI) medium 1640 supplemented with 10% (*v*/*v*) fetal calf serum (Dutscher, Brumath, France).

### 3.5. Determination of Cell Viability

Cells were seeded into a 96-well plate 5 × 10^3^–1 × 10^4^ cells per well. The next day, cells were challenged for 48 h with increasing concentrations of the essential oil and its fractions for 48 h. After the indicated times, cells were washed with PBS and then stained with crystal violet (0.5% *w*/*v*) for 5 min and then rinsed three times with water. Absorbance was read at 540 nm after extraction of the dye with 0.1 M sodium citrate in 50% ethanol. The inhibitory concentrations of 50% (IC_50_) were calculated using a four-parameter nonlinear regression with GraphPad Prism version 6 software (GraphPad Software, La Jolla, CA, USA). The chemotherapeutical drug 5-fluorouracil was used as positive control [40].

## 4. Conclusions

The chemical composition of *T. articulata* trunk bark complete essential oil (HEE) and its fractions (E.1–E.5) is dominated by oxygenated sesquiterpenes (44.4–70.2%). Caryophyllene oxide, carotol and 14-hydroxy-*epi*-(*E*)-caryophyllene were determined as the major components. HEE and its fractions (E.1–E.5) did not exhibit substantial cytotoxic activity against human cell line MDA-MB-231 and SW640.The increasing content of oxygenated sesquiterpenes correlated with an increase of the cytotoxicity.

## Figures and Tables

**Table 1:** Constituents of *T. articulata* trunk bark essential oil (HEE) and its fractions (E.1–E.5) identified by GC/MS.

	Compound	LRI ^a^	Composition (%)						*m/z* ^c^
			HEE	E.1	E.2	E.3	E.4	E.5	
**1**	2-Nonanol	1100	0.1	0.2	- ^b^	-	-	-	45, 69, 144
**2**	Fenchol	1113	0.2	0.4	-	-	-	-	81, 80, 154
**3**	α-Campholenal	1126	0.2	0.3	-	-	-	-	108, 93, 152
**4**	*trans*-Pinocarveol	1141	0.3	0.9	-	-	-	-	92, 70, 152
**5**	*cis*-Verbenol	1142	0.1	0.2	-	-	-	-	94, 109, 152
**6**	*trans*-Verbenol	1143	0.4	1.2	-	-	-	-	91, 109, 152
**7**	Camphene hydrate	1150	0.1	0.4	-	-	-	-	71, 43, 154
**8**	Isoborneol	1156	0.1	0.4	-	-	-	-	95, 41, 154
**9**	Borneol	1168	2.4	4.1	0.3	-	-	0.6	95, 110, 154
**10**	4-Terpineol	1179	0.2	0.6	-	-	-	-	71, 111, 154
**11**	*p*-Cymen-8-ol	1185	1.2	1.4	0.7	0.4	0.4	0.8	43, 135, 150
**12**	α-Terpineol	1191	7.5	10.4	1.3	0.4	0.3	0.9	59, 93, 154
**13**	Myrtenol	1193	1.2	2.5	0.3	-	-	0.2	79, 91, 152
**14**	Verbenone	1205	1.3	1.3	0.7	0.6	0.4	1.1	107, 135, 150
**15**	*trans*-Carveol	1220	0.9	1.2	0.4	-	-	0.3	109, 84, 152
**16**	*cis*-Myrtanol	1251	0.4	0.7	-	-	-	-	41, 69, 154
**17**	*trans*-Myrtanol	1258	0.7	0.9	0.4	-	-	-	42, 69, 154
**18**	Bornyl acetate	1286	0.5	1.1	-	-	-	-	95, 43, 196
**19**	Carvacrol	1298	0.1	0.4	-	-	-	-	135, 91, 150
**20**	Cyclosativene	1369	-	-	0.3	-	-	0.3	161, 105, 204
**21**	Longicyclene	1371	-	-	0.3	-	-	0.5	94, 105, 204
**22**	α-Copaene	1377	0.2	0.6	0.7	0.8	0.5	0.7	161, 119, 204
**23**	Longifolene	1404	1.2	1.4	1.7	1.8	1.1	1.2	161, 94, 204
**24**	β-Caryophyllene	1419	5.5	5.2	2.6	2.9	0.6	-	93, 133, 204
**25**	α-Humulene	1455	1.4	1.6	1.0	1.0	0.3	-	98, 80, 204
**26**	γ-Muurolene	1478	0.2	0.5	0.9	0.9	1.1	1.3	161, 105, 204
**27**	8,9-Dehydrothymol isobutyrate	1480	0.6	-	-	-	-	-	148, 133, 218
**28**	Thymylisobutyrate	1481	3.4	0.1	-	0.3	-	-	135, 150, 220
**29**	Nerylisobutyrate	1489	1.8	0.1	-	-	-	-	69, 93, 208
**30**	α-Muurolene	1499	5.5	6.7	9.1	11.6	11.2	12.5	105, 161, 204
**31**	Modhephen-8-*β*-ol	1508	6.6	0.2	-	-	-	-	189, 119, 204
**32**	*trans*-γ-Cadinene	1514	-	-	-	0.3	0.3	0.4	161, 105, 204
**33**	*cis*-Calamenene	1523	-	-	0.7	-	0.7	0.8	159, 160, 202
**34**	δ-Cadinene	1524	0.9	1.0	-	1.1	-	-	161, 134, 204
**35**	α-Calacorene	1543	-	-	-	0.3	-	-	157, 142, 200
**36**	Elemol	1550	0.8	1.4	1.3	0.8	0.6	0.5	59, 93, 222
**37**	Dodecanoicacid	1567	-	-	-	-	-	2.7	73, 60, 200
**38**	Palustrol	1569	-	-	0.8	0.7	-	0.5	41, 55, 222
**39**	Neryl 2-methylbutyrate	1575	2.5	0.1	-	-	-	-	69, 41, 238
**40**	Caryophyllene oxide	1581	13.1	25.2	26.6	20.5	20.2	21.9	43, 41, 220
**41**	Geranyl 2-methylbutyrate	1586	4.9	0.2	-	-	-	-	69, 41, 238
**42**	1-Hexadecene	1592	1.8	-	-	-	-	-	43, 55, 224
**43**	*cis-*Arteannuicacid	1593	4.1	-	1.2	-	-	-	121, 119, 234
**44**	*n*-Hexadecane	1600	-	-	-	-	0.6	-	57, 43, 226
**45**	Carotol	1602	9.2	12.0	20.3	20.7	21.2	15.1	161, 204, 222
**46**	Humulene epoxide II	1607	2.6	4.0	7.2	6.1	6.2	5.4	109, 67, 220
**47**	1-*epi*-Cubenol	1629	-	-	-	0.8	0.6	-	119, 41, 222
**48**	γ-Eudesmol	1631	-	-	-	0.7	0.8	-	189, 204, 222
**49**	Caryophylla-4(14),8(15)-dien-5-ol	1637	1.6	1.2	2.0	2.0	1.8	1.4	136, 91, 220
**50**	T-Cadinol	1641	1.5	1.1	2.1	1.9	2.2	1.3	161, 43, 222
**51**	T-Muurolol	1642	1.7	0.9	1.9	2.3	2.7	1.8	95, 121, 222
**52**	14-Hydroxy-9-*epi-(E)*-caryophyllene	1665	3.2	2.7	6.8	9.6	13.1	15.5	91, 93, 220
**53**	Cadalene	1673	1.3	1.2	2.5	3.4	3.9	3.2	183, 168, 198
**54**	1-Octadecene	1793	1.7	-	-	-	-	-	43, 41, 252
Oxygenated monoterpenes			31.0	28.9	4.1	1.7	1.1	3.9	
Sesquiterpene hydrocarbons			16.2	18.2	19.8	24.1	19.7	20.9	
Oxygenated sesquiterpenes			44.4	48.7	70.2	66.1	69.4	63.4	
Nonterpene derivatives			3.6	0.2	0.0	0.0	0.6	2.7	
Total identified			95.2	96.0	94.1	91.9	90.8	90.9	

^a^ Linear retention indices calculated on a DB5 capillary column; ^b^ not detected; ^c^ two main peaks and the molecular one.

**Table 2:** IC_50_ (µg/mL) values for *T. articulata* trunk bark complete essential oil (HEE) and its fractions (E.1–E.5) towards MDA-MB-231 and SW620 cell lines.

IC_50_	5-FU	HEE	E.1	E.2	E.3	E.4	E.5
MDA-MB-231	2.2	83.0	96.5	90.7	85.9	91.8	100.0
SW620	0.3	25.7	37.8	26.2	43.1	57.2	96.5

## Data Availability

Not applicable.
